# A superconducting adiabatic neuron in a quantum regime

**DOI:** 10.3762/bjnano.13.57

**Published:** 2022-07-14

**Authors:** Marina V Bastrakova, Dmitrii S Pashin, Dmitriy A Rybin, Andrey E Schegolev, Nikolay V Klenov, Igor I Soloviev, Anastasiya A Gorchavkina, Arkady M Satanin

**Affiliations:** 1 Faculty of Physics, Lobachevsky State University of Nizhni Novgorod, 603950 Nizhny Novgorod, Russia; 2 Skobeltsyn Institute of Nuclear Physics, Lomonosov Moscow State University, 119991 Moscow, Russia; 3 Moscow Technical University of Communication and Informatics (MTUCI), 111024 Moscow, Russia; 4 Faculty of Physics, Lomonosov Moscow State University, 119991 Moscow, Russia; 5 Higher School of Economics, Russia National Research University, 101000 Moscow, Russia; 6 Federal State Unitary Enterprise All-Russia Research Institute of Automatics named after N.L. Dukhov, 101000 Moscow, Russia

**Keywords:** Josephson junction, quantum neuron, quantum-classical neural networks, superconducting quantum interferometer

## Abstract

We explore the dynamics of an adiabatic neural cell of a perceptron artificial neural network in a quantum regime. This mode of cell operation is assumed for a hybrid system of a classical neural network whose configuration is dynamically adjusted by a quantum co-processor. Analytical and numerical studies take into account non-adiabatic processes as well as dissipation, which leads to smoothing of quantum coherent oscillations. The obtained results indicate the conditions under which the neuron possesses the required sigmoid activation function.

## Introduction

**Figure 1:**
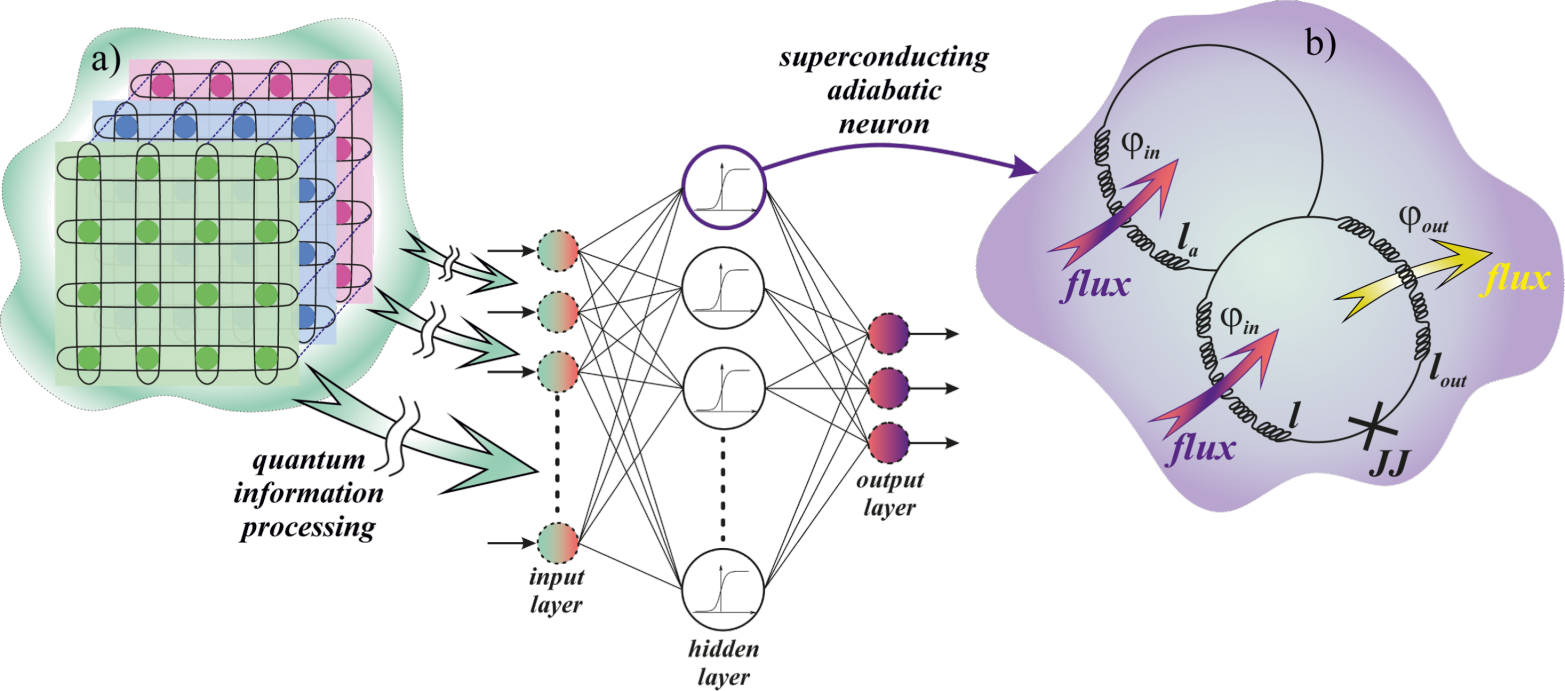
(a) Sketch of a flexible hybrid system consisting of a classical ANN having its configuration (synaptic weights) dynamically adjusted by a quantum co-processor. (b) Schematic representation of the *S*_Q_ neuron providing nonlinear magnetic flux transformation.

The implementation of machine learning algorithms is one of the main applications of modern quantum processors [1–9]. It has been shown that a relatively small quantum circuit may be capable of searching for a large number of synaptic weights of an artificial neural network (ANN) [10–13]. The rate of the weight adjustment is an important parameter that determines the possibility of the ANN dynamic adaptation. Such tunability is required when working with rapidly changing content. The corresponding information flow naturally arises, for example, within the framework of novel telecommunication paradigms, such as software-defined radio [14–15] implying the change of signal frequency and modulation. An efficient architecture of a flexible hybrid system requires a close spatial arrangement of the classical ANN with its control quantum co-processor, see Figure 1a. Superconductor technology is a promising platform for such a solution since both superconducting quantum machine learning circuits [16–22] and superconducting ANNs [23–37] are rapidly developed nowadays.

Robust implementation of the considered quantum-classical system would benefit from the utilization of a single technology suitable for superconducting qubits. In this case, the classical part can operate in an adiabatic mode ensuring minimal impact on quantum circuits. However, quantum effects, in turn, can significantly affect the operation of neuromorphic elements. In this work, we account for this by considering the neuron cell operation in a quantum regime. We investigate the dynamics of this cell in search of conditions that provide the required sigmoid activation function (conversion of the input magnetic flux into the average output current), suitable for the operation of the ANN as a perceptron [4]. The studied cell is called a quantum neuron or *S*_Q_ neuron. Its closest analogue is the flux qubit used by D-Wave Systems in quantum annealers [38–41].

An important incentive for this work are the previously obtained results on classical adiabatic neurons with extremely small energy dissipation [42–45]. We especially note the demonstrated possibility of the adiabatic evolution of the state for a neuron in a multilayer perceptron with Josephson junctions without resistive shunting [46]. It is precisely such a heterostructure without resistive shunting that is used in the implementation of a quantum neuron based on a flux qubit.

The article is organized as follows. First, we present the scheme of the proposed quantum neuron and also investigate the spectrum of the Hamilton operator for such a system. Next, on the basis of the numerical solution of the Schrödinger equation, we investigate dynamic processes in a quantum neuron. We pay special attention to the analysis of the activation function of the cell for two main modes (with one and two minima of the potential energy of the system). We use Wigner functions for a visual interpretation of the dynamics of the neuron. The range of the operating parameters for the proposed neuron circuit under the action of unipolar magnetic flux pulses is found. Finally, the influence of the dissipation on the features of the dynamic processes and characteristics of the cell is revealed.

## Methods

### Neuron model and basic equations

A single-junction superconducting interferometer with normalized inductance *l*, a Josephson junction without resistive shunting (JJ), an additional inductance *l*_a_, and an output inductance *l*_out_ (see Figure 1b) are the basis of the quantum neuron. This circuit has been presented before as a classic superconducting neuron for an adiabatic perceptron [42,46].

The classical dynamics of the system under consideration is described using the equation for the dynamics of the Josephson phase:


[1]

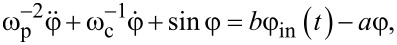



where the coefficients are determined by the expressions


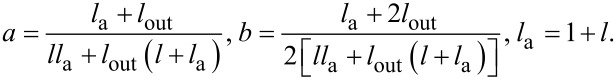


These coefficients were introduced in [46] when considering the classical mode of this system. Inductances are normalized to (2π*I*_c_/Φ_0_), where *I*_c_ is the critical current of the Josephson junction and Φ_0_ is the magnetic flux quantum. The inertial properties of the system are due to the junction capacitance, which, along with the critical current *I*_c_, determines the plasma frequency of the JJ,


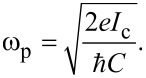


In this case, the dissipative properties of the system are determined by the Josephson characteristic frequency ω_c_ = 2*eRI*_c_ℏ (here, *R* and *C* are the normal state resistance and capacitance of the Josephson junction, respectively).

Dynamic control of the system states is carried out by a changing external magnetic flux, φ_in_(*t*), normalized to the magnetic flux quantum Φ_0_:


[2]

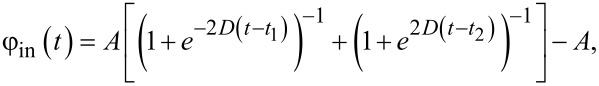



where *A* is the normalised amplitude of the external action, *t*_1_ and *t*_2_ = 3*t*_1_ are the characteristic rise/fall times of the control signal, whose steepness is determined by the parameter *D*. The phase of the Josephson junction, φ, obeys Equation 1. The activation function of the neuron is determined by the dependence of the output current *i*_out_ on the input flux φ_in_:


[3] $$i_{\text{out}}=\frac{\varphi_{\text{in}}-2l_{\text{a}}i}{2\left(l_{\text{a}}+l_{\text{out}}\right)},\;i=b\varphi_{\text{in}}-a\varphi .$$

### Spectrum of the neuron Hamiltonian

The quantum regime manifests itself through a discrete spectrum of allowed values for the total energy of the system. The characteristic gaps in the spectrum of the effective Hamiltonian are significantly larger than the thermal smearing in the studied case. Also, the level broadening due to the influence of the environment is relatively small. The described features affect the neuron ability to non-linearly transform the magnetic signal. In order to describe the quantum mechanical behavior of the system (Equation 1), we start from the case of a Josephson junction with a large shunted resistance (ω_c_^−1^→0). In this case, Equation 1 can be interpreted as the equation of motion for a particle with mass *M* = ℏ^2^/2*E*_c_ (charge energy *E*_c_ = (2*e*)^2^/2*C*) in potential


[4] $$\begin{array}{c} U\left[\varphi ,\varphi_{\text{in}}\left(t\right)\right]=E_{J}\frac{{\left[b\varphi_{\text{in}}\left(t\right)-a\varphi \right]}^{2}}{2a}+E_{J}\left(1-\cos\varphi \right),\\ \text{with }E_{J}=\frac{I_{\text{c}}\Phi_{0}}{2\pi }. \end{array}$$

The dynamics of the system is governed by the Hamilton function,


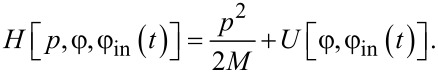


The canonical quantization procedure leads to the Hamiltonian:


[5]

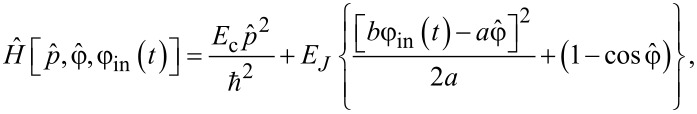



where the operators 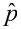 and 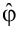 obey the commutative relation 
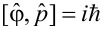
.

The form of the potential from Equation 4 in each moment of time, and hence the dynamic behavior of the system, is determined by the physical parameters of the circuit shown in Figure 1. There is a range of inductance values where the potential profile from Equation 4 has a double-well shape under the action of the input flux (Equation 2). Their values can be obtained from solution of the transcendental equation


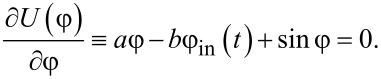


The potential has more than one extremum in the case that *a <* 1 and ,therefore,


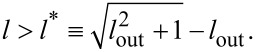


Note that for the classical regime the sigmoidal shape of the activation function is possible only when l < l^*^ [46].

One of the goals of this work is to determine the parameters of the adiabatic switching of the quantum neuron for l < l^*^ (single-well mode) and l > l^*^ (double-well mode). Within the adiabatic approach it is possible to numerically solve the time-independent Schrödinger equation (see Appendix 1) for each moment of time to find “instantaneous energy levels”, *E_n_*(*t*), and “instantaneous wave functions” of the system, ψ*_n_*(φ, *t*):


[6]

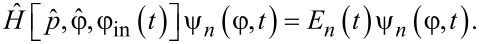



**Figure 2:**
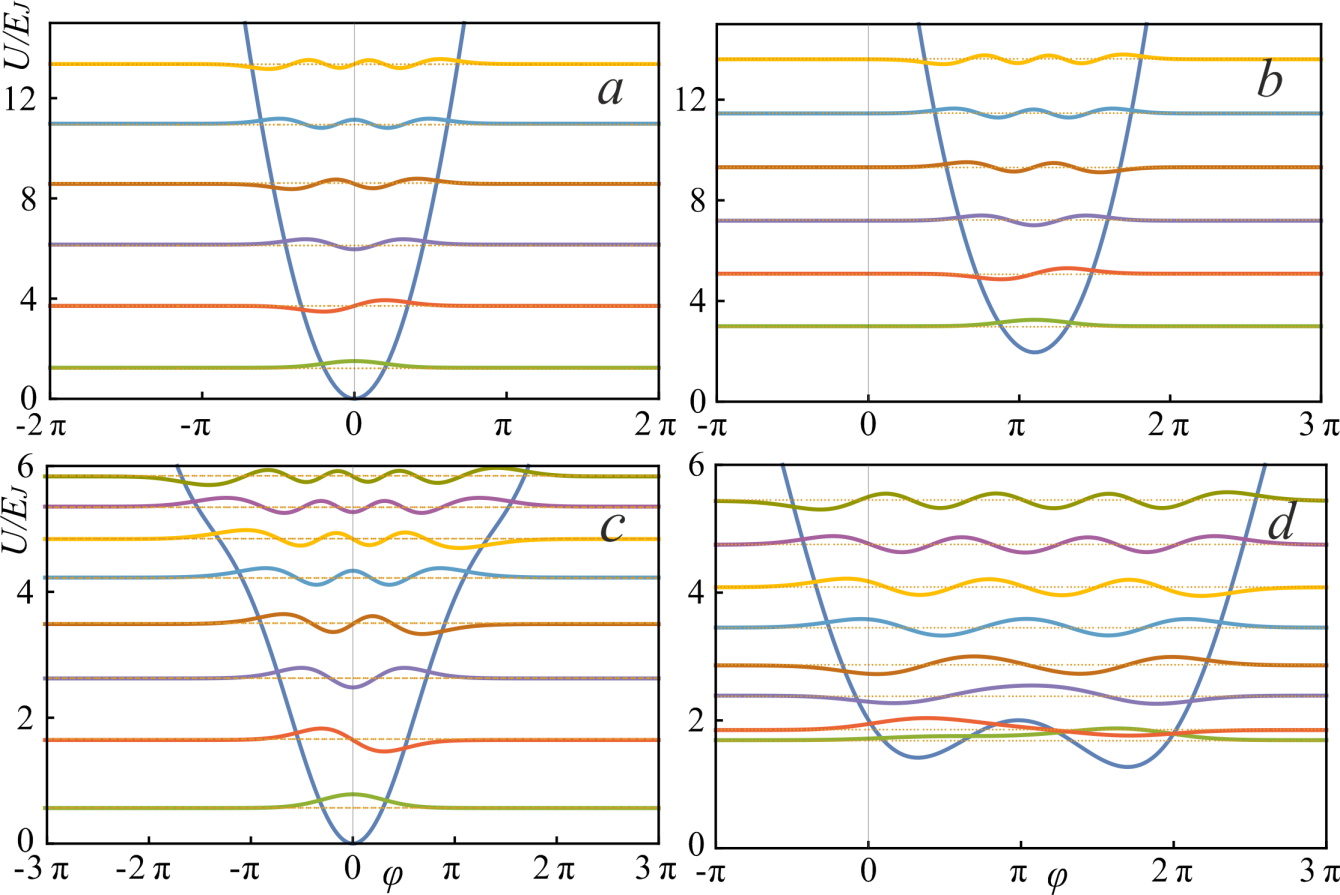
The energy spectrum and adiabatic (instantaneous) wave functions are represented at the initial time *t* = 0 (a, c) and at the rise of the applied flux, *t*_1_ = 500 (b, d) for the inductance values *l* = 0.1 (a, b) and *l* = 2.5 (c, d). The parameters of the system and the input magnetic flux are: *E_C_* = 0.5*E_J_*, *l*_a_ = *l* + 1, *l*_out_ = 0.1, *D* = 0.008, *A* = 4π.

Figure 2 demonstrates the spectrum of instantaneous energy levels and wave functions of the system at the initial moment of time (Figure 2a,c) and at the moment *t*_1_, when the input magnetic flux (Equation 2) is equal to φ_in_ = 2π (Figure 2b,d). Note that for the case l < l^*^ (Figure 2a,b), the form of the potential can be approximated by a parabolic function (single-well mode). The symmetry of the potential under external influence does not change, and only a shift in the energy levels with preservation of the interlevel distance is observed during the rise/fall periods of the signal. A different behavior is observed for l > l^*^ where during the rise/fall periods of the signal, a double-well potential appears (Figure 2d). Here, the two lowest close energy levels are separated by an energy gap from the rest of the level structure. This resembles the formation of the flux qubit spectrum [47].

## Results and Discussion

### Dynamics of the quantum neuron without dissipation

The dynamics (evolution of the states of the system, Ψ(*t*)) of the quantum neuron (Equation 5) is associated with the nonlinear transformation of the input magnetic flux (Equation 2). We describe it using the time-dependent Schrödinger equation:


[7]

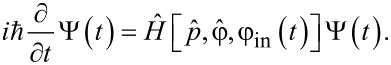



Eigenvectors of the system are found by numerical solution of Equation 7 (see details in Appendix 2). Thereafter, from the evolution of average values of the phase and current operators we found the transfer characteristic *i*_out_(φ_in_) of the *S*_Q_ neuron Equation 3, that is, its activation function. Let us explain the idea of our calculations. We assume that the system is initialized at the initial moment of time. At cryogenic temperatures (millikelvin range) the system states are localised at lower energy levels.

According to Equation 3, the dependence of the average value of the output current *i*_out_ on the input magnetic flux φ_in_ is calculated:


[8]

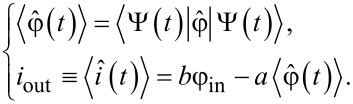



We use the Wigner functions in order to visualize the adiabatic dynamics in the “phase-conjugate momentum” space [48]. This function is determined by the Fourier transform of a bilinear combination of the wave functions:


[9]

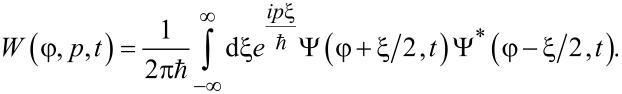



The wave function Ψ(φ,*t*) can be expanded in terms of the instantaneous eigenvectors ψ*_n_*(φ,*t*):


[10]

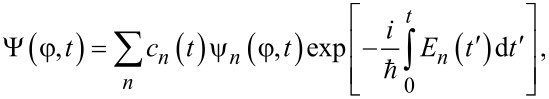



where the coefficients *c_n_*(0) are determined from the initial conditions for the wave function Ψ(φ,0). Changes of the coefficients *c_n_*(*t*) in time are determined by the system of *N* coupled equations


[11]

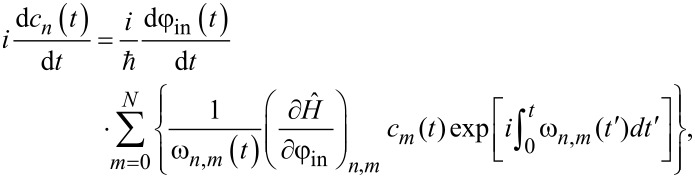



where the time-dependent matrix elements appear. Their rate of change is given by 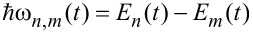. Note that if the adiabaticity condition,


[12]

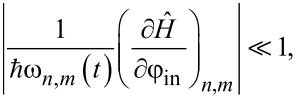



is satisfied for pairs of levels, then transitions between them become improbable.

We consider the case where only the two lowest levels are taken into account. In this case, the remaining energy levels lie noticeably higher than the selected doublet. In addition, adiabaticity conditions (Equation 12) should be satisfied. When these conditions are met, the following expression can be written to approximate the wave function:


[13]

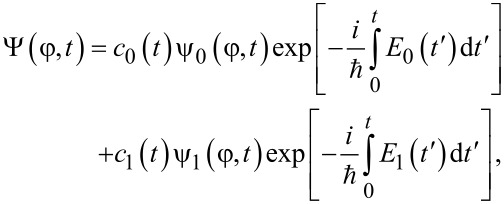



and we can get the expression for the Wigner function:


[14] $$\begin{array}{c} W\left(\varphi ,p,t\right)={\left|c_{0}\left(t\right)\right|}^{2}K_{0,0}\left(\varphi ,p,t\right)+{\left|c_{1}\left(t\right)\right|}^{2}K_{1,1}\left(\varphi ,p,t\right)\\ +c_{0}\left(t\right)c_{1}^{*}\left(t\right)K_{0,1}\left(\varphi ,p,t\right)\exp\left[i\int_{0}^{t}\omega_{0,1}\left(t^{\prime }\right)\text{d}t^{\prime }\right]\\ +c_{1}\left(t\right)c_{0}^{*}\left(t\right)K_{1,0}\left(\varphi ,p,t\right)\exp\left[-i\int_{0}^{t}\omega_{0,1}\left(t^{\prime }\right)\text{d}t^{\prime }\right], \end{array}$$

where


[15] $$K_{n,m}\left(\varphi ,p,t\right)=\frac{1}{2\pi }\int_{-\infty }^{\infty }\text{d}\xi e^{ip\xi }\psi_{n}\left(\varphi +\xi /2,t\right)\psi_{m}^{*}\left(\varphi -\xi /2,t\right).$$

Further we demonstrate two effects in this approximation: (1) One can construct a superposition of the basis states and observe the manifestation of the interference of quantum states in the oscillations of the output characteristic; (2) there are oscillations of the output characteristic due to the influence of nonadibaticity.

### Single-well potential

**Figure 3:**
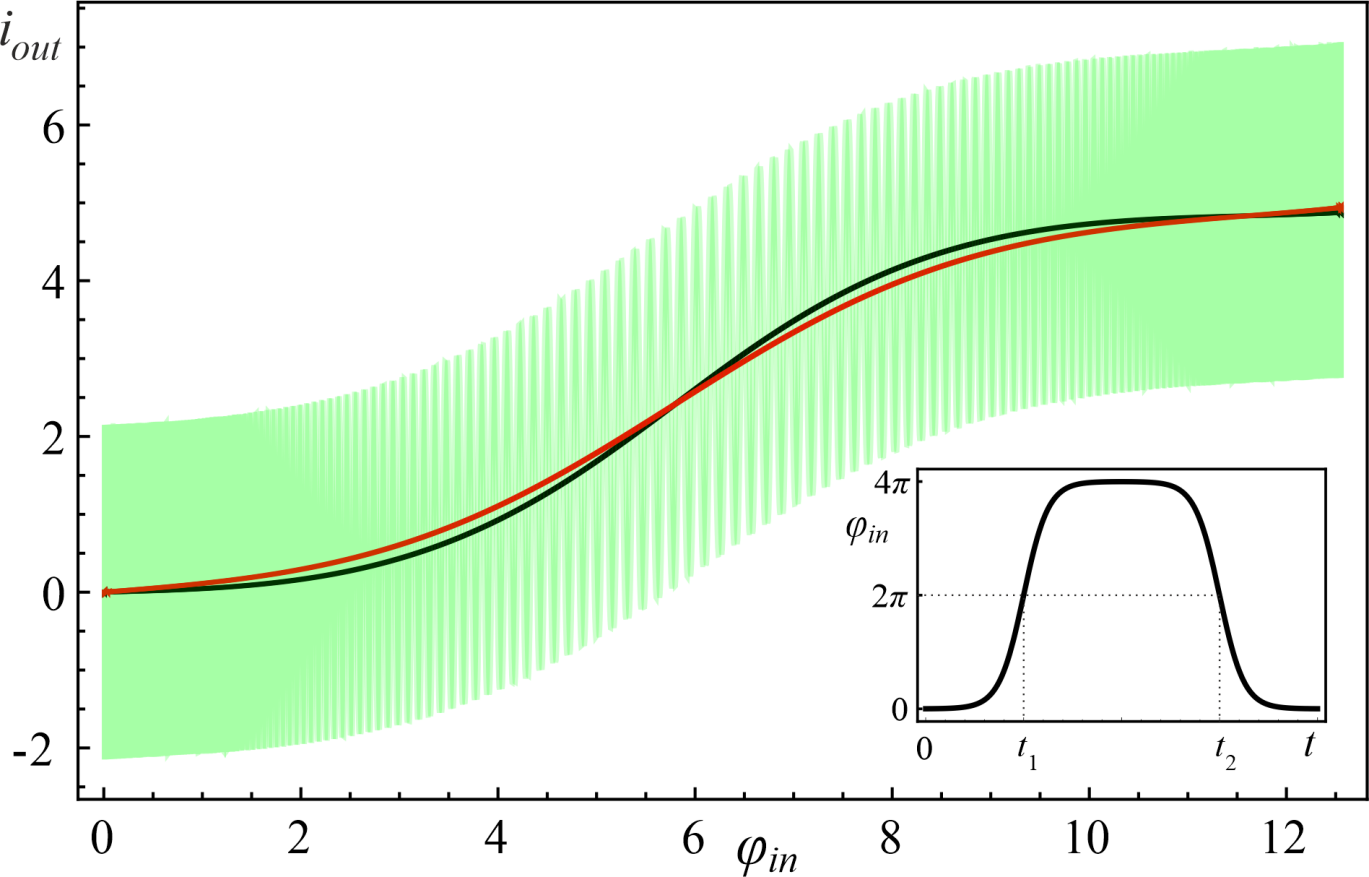
The neuron activation functions for *l* = 0.1 and different initial states: The black curve corresponds to the ground initial state ψ_0_(φ,0), the red curve to the first excited state ψ_1_(φ,0), and green curve corresponds to the superposition of states (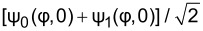. Parameters of the input magnetic flux are *D* = 0.008, *A* = 4π, and *t*_1_ = 500.

Figure 3 demonstrates the calculated activation functions of the *S*_Q_ neuron operating in the quantum regime in single-well mode (l < l^*^) for three different initial states of the system.

Numerical analysis has shown that the activation functions for the quantum neuron, initialised in the basic states, takes a sigmoidal shape (black and red curves in Figure 3). This is in a good agreement with the classical regime of operation [46].

Note that when the input flux (Equation 2) changes from 0 to 4π, the phase φ on the Josephson junction changes from 0 to 2π and vice versa.

The complete coincidence of the two paths of the system evolution occurs with a significant increase in the rise time “↑” (φ = 0→2π) and the fall time “↓” (φ = 2π→0) of the input signal. For the superposition of the basic states, as seen in Figure 3, oscillations are observed in the shape of the activation function. In this regard, for clarity of interpretation of the obtained results of the quantum dynamics, we consider the evolution of the system in the phase space.

**Figure 4:**
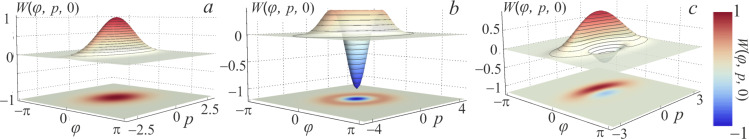
The Wigner functions *W*(φ, *p*, *t* = 0) of the considered system initialized at the initial moment of time *t* = 0 (a) in the ground state ψ_0_(φ,0), (b) in the first excited state ψ_1_(φ,0) and (c) in the superposition of the lowest states 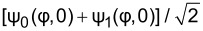 for *l* = 0.1. Other parameters are similar to those shown in Figure 3.

**Figure 5:**
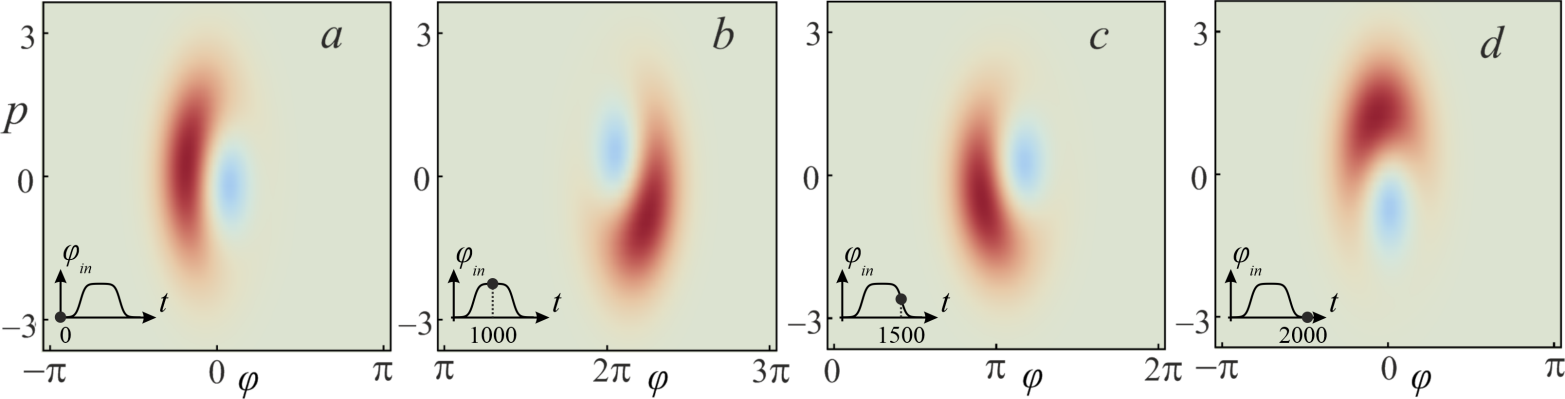
The evolution of the Wigner function under the influence of the input flux φ_in_ for the *S*_Q_ neuron initialized in the superposition state 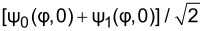 at the moments *t* = 0 (absence of φ_in_) (a); *t* = 1000 (the plateau of φ_in_) (b); *t* = 1500 (the middle of the decreasing branch of φ_in_) (c); *t* = 2000 (absence of φ_in_) (d). The remaining parameters are similar to those shown in Figure 3.

If the adiabaticity condition (Equation 12) is satisfied and the system was initially at the lowest level |*c*_0_(0)|^2^ = 1 (see Figure 4a), then the dynamics of the Wigner function reflects the distribution in phase and conjugate momentum related to this level. Similar reasoning can be given for the case when the first excited level (Figure 3b) is populated. Here, the center of the probability density |Ψ(φ,*t*)|^2^ and the distribution of the Wigner function (Figure 4a,b) shift smoothly, from φ = 0 to 2π, when the cell is exposed to the input magnetic flux. The system remains localized in the initial state, and as a result, the activation function takes a sigmoidal form without any oscillations (black and red curves in Figure 3). If the system is initialised in the superposition of the lowest states (Figure 4c) then the interference term in the Wigner function emerges, see the last two terms in Equation 14. This is expressed as oscillations on the Wigner function between the maximum (red area) and minimum (blue area), see Figure 5. Coherent oscillations on the current–flux dependence are also the evidence of this phenomenon (see the green curve in Figure 3).

### Double-well potential

**Figure 6:**
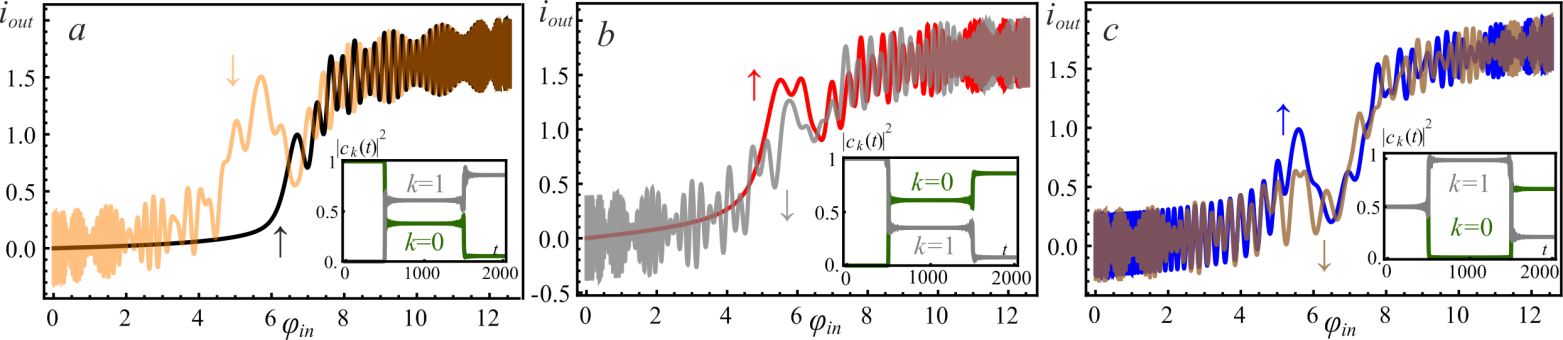
The activation functions of the neuron with *l* = 2.5 initialized (a) in the ground state, see the black “↑” and orange “↓” curves; (b) in the first excited state, see the red “↑” and gray “↓” curves; (c) in the superposition of the basis states, see the blue “↑’ and brown “↓” curves. Input flux parameters are *D* = 0.008, *A* = 4π, *t*_1_ = 500. The symbol “↑” corresponds to the rise branch of φ_in_ = 0→4π, the “↓” symbol corresponds to the fall branch of φ_in_ = 4π→0. The inserts show the time-dependent evolution of the populations |*c_k_*(*t*)|^2^ of the ground state, *k* = 0, and the first excited, *k* = 1, state of the system.

For the double-well potential, when l > l^*^, the problem of quantum dynamics and the formation of the sigmoidal activation function have also been studied. We start with the parameters of the input flux as presented in Figure 3. Numerical simulations demonstrate a distortion of the sigmoidal form of the activation function even when the *S*_Q_ neuron is initialized in the ground state, see Figure 6.

**Figure 7:**
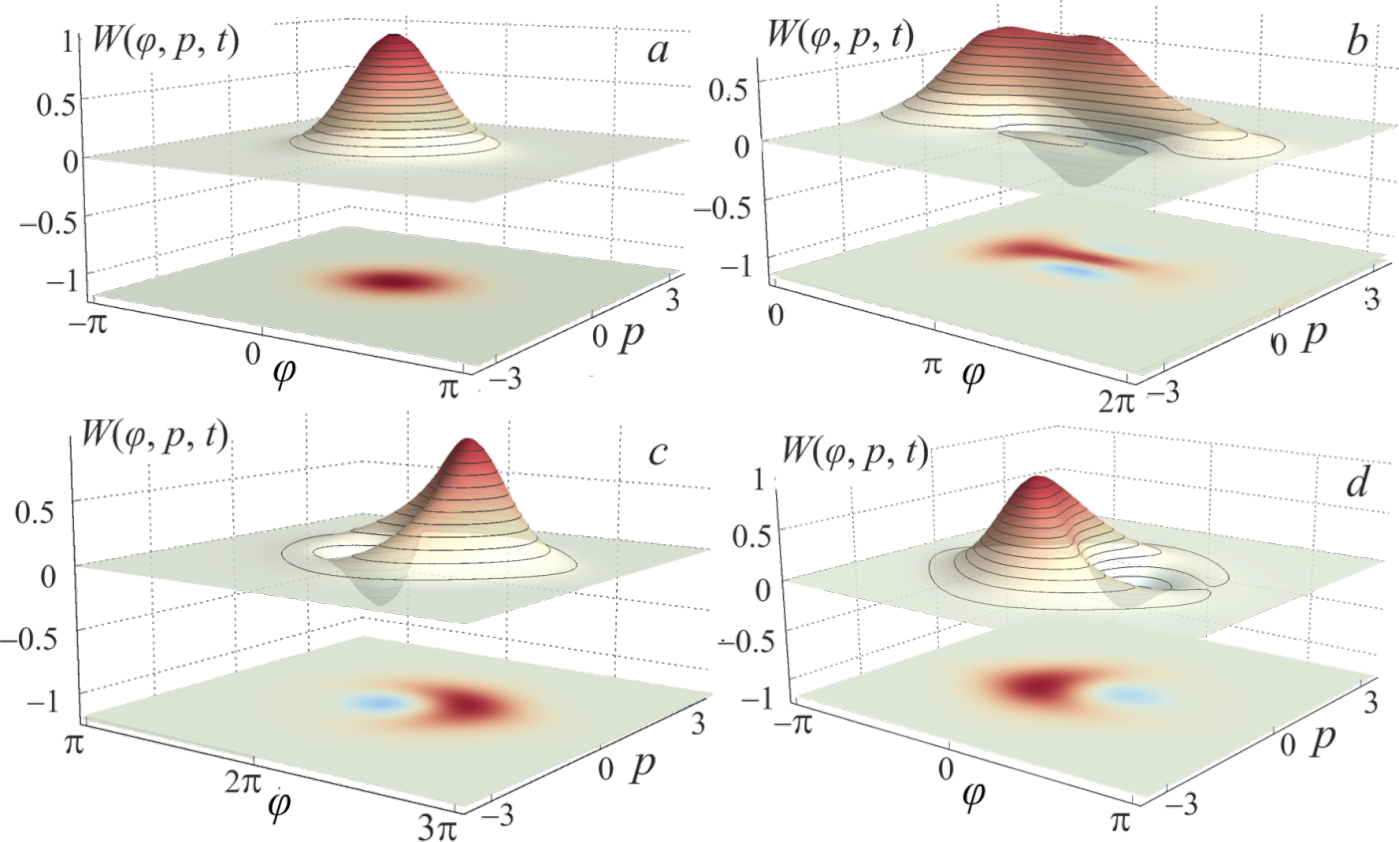
Evolution of the Wigner function of the *S*_Q_ neuron with *l* = 2.5 initialized in the ground state under the action of the input flux φ_in_ at the moments *t* = 0 (absence of φ_in_) (a); *t* = 500 (the middle of the increase of φ_in_) (b); *t* = 1000 (the plateau of φ_in_) (c); *t* = 2000 (absence of φ_in_) (d). The input flux parameters are equal to those shown in Figure 6.

In the process of evolution, a significant rearrangement occurs in the spectrum of energy levels (anti-crossing between the ground and the first excited levels) during the formation of a double-well potential (see Figure 2). This corresponds to the rise period of the signal along the path φ = 0→2π. Note that, in this case, the adiabaticity condition (Equation 12) is violated. This is a consequence of the increase in the input flux φ_in_, which leads to the excitation of the overlying states. In this case, the system ceases to be localized in the initial state, which is clearly shown in Figure 7 during the evolution of the Wigner function in the phase space. It can be seen that the system evolves adiabatically from the ground state until reaches φ_in_ = 2π, when a double-well potential profile (Equation 4) is formed. In this case, the rate of change of the potential exceeds the rate of state localisation. Due to the tunneling effect, the wave function is redistributed from the left to the right local minimum of the potential profile (see Figure 2). Figure 7b,c clearly shows that the Wigner function has negative values due to the formation of a superposition state during evolution (see also the insets in Figure 6 for the population coefficients |*c*_0_(*t*)|^2^ and |*c*_1_(*t*)|^2^ for basis levels). Because of this, the activation function in Figure 6 exhibits oscillations associated with the interference of the wave functions. These oscillations are more irregular than the ones in Figure 3 (see the green curve). This is due to the occurrence of interference phase effects of a larger number of states participating in the superposition corresponding to the violation of the adiabaticity condition (Equation 12).

**Figure 8:**
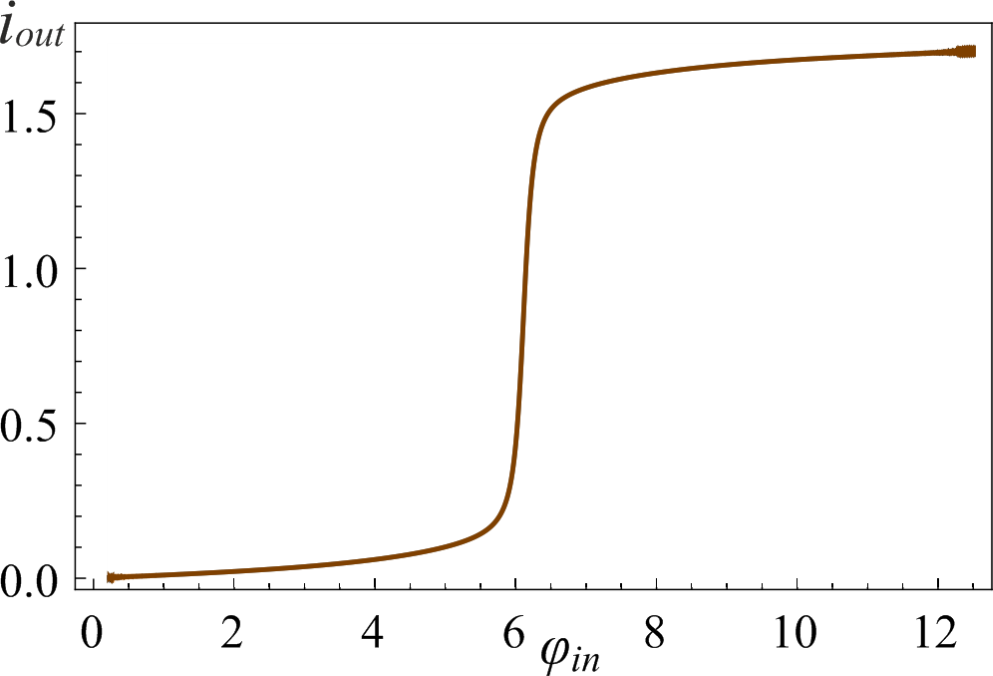
The activation function of the neuron with *l* = 2.5 initialised at *t* = 0 in the ground state. Here the parameters are *D* = 0.0002, *A* = 4π, and *t*_1_ = 10000.

Note that if the rate of the potential changes is less than the rate of the localised state movement and the adiabaticity condition (Equation 12) is satisfied, we can obtain the sigmoidal activation function even in a double-well potential (see Figure 8). In this case, there is a good match between the forward “↑” and the backward “↓” characteristics of the *S*_Q_ neuron.

### Activation function of the quantum neuron

**Figure 9:**
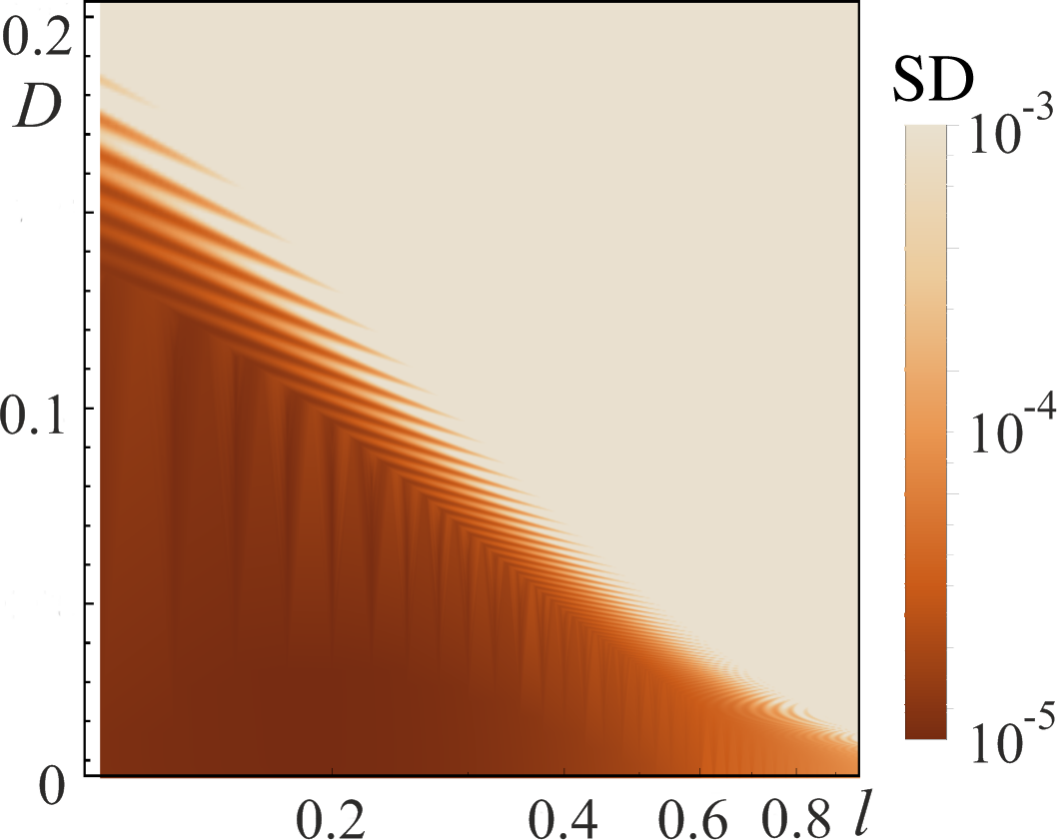
The value of the square of the standard deviation, SD, of the *S*_Q_ neuron activation function from the mathematical sigmoid (Equation 16) for different inductance *l* values and rise/fall rates, *D*, of the input flux φ_in_(*t*). The horizontal axis is in logarithmic scale. At the initial moment, the system was initialized in the ground state. The parameters of the system and the input flux are as follows: *A* = 4π, *l*_a_ = *l* + 1, *l*_out_ = 0.1.

We also study the quality of approximation of the neuron activation function by the sigmoidal function for different parameters of the cell (in the framework of the adiabaticity conditions). The approximation function is:


[16] $$\sigma \left(\varphi_{\text{in}}\right)=\frac{p_{1}}{1+e^{-p_{2}\varphi_{\text{in}}+p_{3}}}+p_{4},$$

where *p_i_* are the parameters of the numerical approximation. Our goal is to compare the ideal activation function σ(φ_in_) and the activation function of the considered cell *i*_out_(φ_in_). We use the square of the standard deviation, SD, for this purpose:


[17] $$\text{SD}=\text{Dis}\left[{\left(\sigma \left(\varphi_{\text{in}}\right)-i_{\text{out}}\left(\varphi_{\text{in}}\right)\right)}^{2}\right],$$

where Dis[(…)] means the dispersion of a data set. Analysis of Figure 6 and Figure 8 allows us to conclude that the parameters affecting the activation function shape are primarily the rise/fall rate of the signal *D* (see Equation 2) and the inductance value *l*, which determines the shape of the potential profile. In this regard, we obtain the plane of parameters SD(*l*, *D*), presented in Figure 9, where the color indicates the value of the square of the standard deviation from the “ideal sigmoid”. The area with SD *<* 0.0001 (area inside the dark zone in Figure 9) corresponds to the formation of the sigmoid activation function of the required form.

From the analysis of Figure 9, it can be concluded that the higher the value of the inductance *l*, the slower the process of adiabatic switching of the quantum neuron. For superconducting circuit parameters *I*_c_ = 0.35 μA, *C* =10 fF, ω_p_ ≈ 10^11^ s^−1^, the adiabatic switching time is approx. 5 ns for *l* = 0.1 (see Figure 3, the regime without oscillations) and approx. 100 ns for *l* = 2.5 (see Figure 8).

### Influence of dissipation effects on the quantum neuron dynamics

In the classical regime, the dissipation mechanism in the neuron has been considered using the Stewart–McCumber model [49]. To take into account the dissipation in a quantum system, we “place” it in a bosonic bath. For further analysis, we use a linear model of the interaction between the quantum neuron and the bath:


[18]

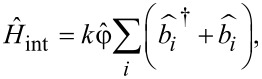



where 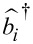 and 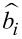 are creation and annihilation operators of the *i*-th bosonic mode, and *k* is the coupling constant. With an adiabatic change of the input flux, the *S*_Q_ state can be described in terms of the instantaneous eigenbasis ψ*_n_*(φ,*t*), see Equation 6, using a density matrix:


[19] $$\rho \left(t\right)=\sum_{m,n}\rho_{mn}\left(\phi ,t\right)|\psi_{m}\left(\phi ,t\right)\rangle \langle \psi_{n}\left(\phi ,t\right)|.$$

Under the Born–Markov approximation, dissipative dynamics is described by the generalized master equation for the density matrix [50]. Furthermore, by keeping only the secular terms and using the random phase approximation, we reduced it to the Pauli master equation (we present the results of modeling for the generalized master equation with and without the secular approximation in Appendix 3):


[20]

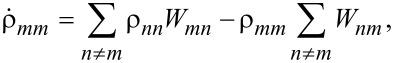



where the dots denote a differentiation by normalized time, *W_mn_* is the transition rate from the state *n* to *m* given by the expression


[21]

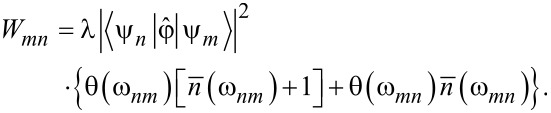



Here λ = (2π*gk*^2^)/ℏ^2^ is the renormalized coupling constant, θ is the Heaviside step function,


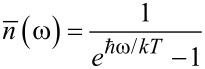


is the Planck distribution, and *g* is the density of bosonic modes, which is supposed to be constant. Under adiabatic approximation, the transition rates *W_mn_* between the neuron states are calculated in the instantaneous eigenbasis. Numerical simulations are performed for the temperature of the bosonic thermostat at *T* = 50 mK.

**Figure 10:**
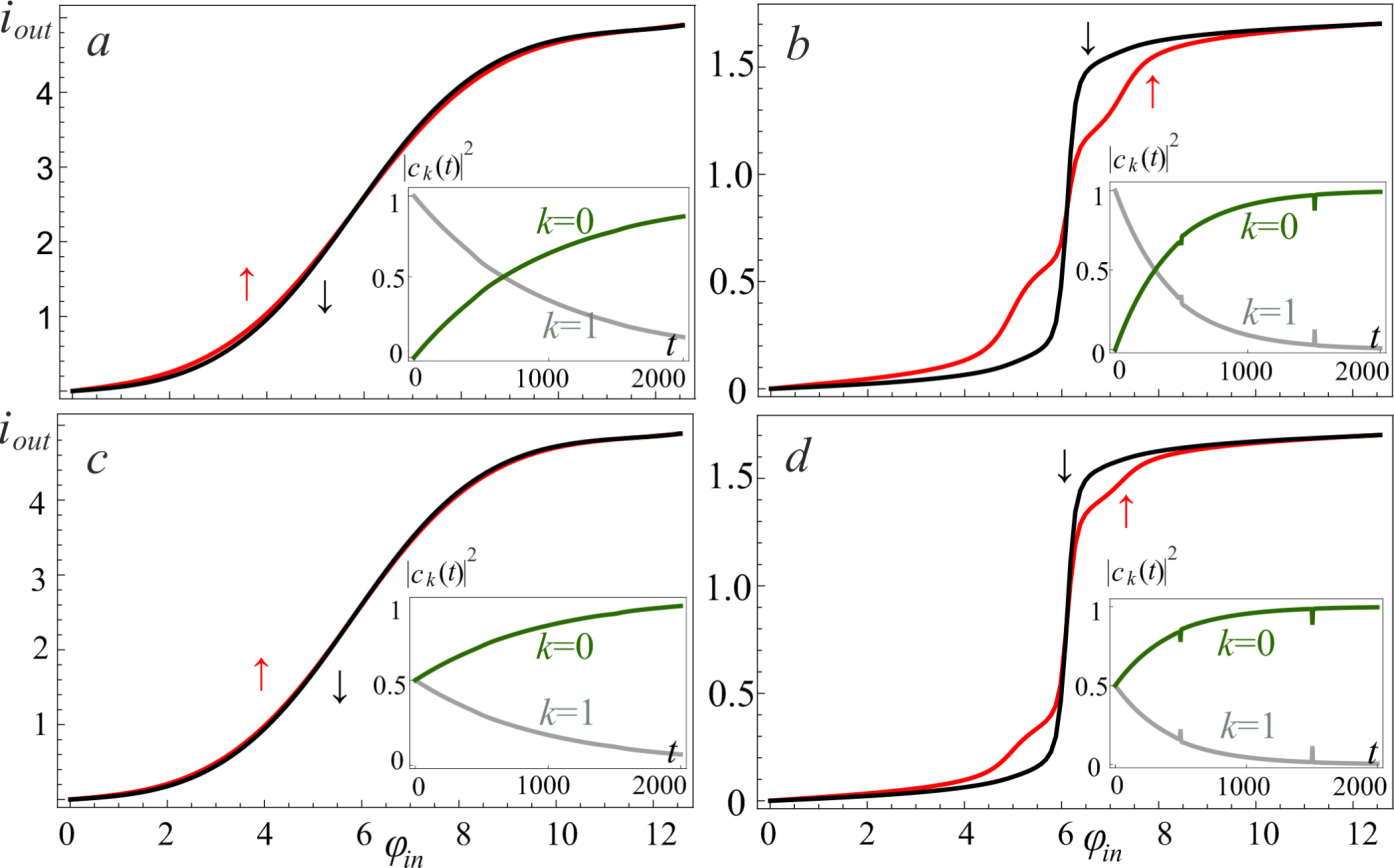
The neuron activation function for *l* = 0.1 (a, c) and *l* = 2.5 (b, d) when the cell is initialized in the first excited level (a, b) and in the superposition of two basic states (c, d). The input flux parameters are as follows: *D* = 0.008, *A* = 4π, *t*_1_ = 500; the renormalized coupling constant λ = 0.005. The insets present the corresponding populations |*c_k_*(*t*)|^2^ of the ground state, *k* = 0, and the first excited, *k* = 1, energy levels.

We investigated the relaxation of the excited states for both the single-well (l < l^*^, Figure 10a,c) and double-well (l > l^*^, Figure 10b,d) potential shapes. The key result is the suppression of the oscillations of the activation function for the neuron initialized in a superposition of two basic states. The dynamics of changes in the populations |*c_k_*(*t*)|^2^ of the energy levels for this case is shown in the insets of Figure 10 (see Figure 6 for comparison). This relaxation takes the full cycle of switching of the input flux (
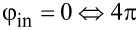
) due to dissipative processes.

In Figure 10b,c there is an obvious suppression of the oscillations on the activation function, which were observed due to the anti-crossing of the energy levels in the double-well potential. In addition, coherent oscillations on the activation function of the neuron (see Figure 3 and Figure 6c) arising during evolution from the superposition state are also smoothed out. Previously, these oscillations were associated with the interference of the phases of the *S*_Q_ states. However, the possible dispersion of the initial phases makes the activation function to be sigmoidal due to the averaging over random phases, see Figure 10c,d.

## Conclusion

We have shown that an adiabatic superconducting neuron of a classical perceptron, under certain conditions, retains the sigmoidal shape of the activation function in the quantum regime (when the spectrum of allowed energy values is discrete). Moreover, the sigmoidal transformation of the applied magnetic flux into the average output current can be obtained both for single-well and double-well potential energies of the cell. The influence of the initial quantum state of the neuron on the shape of the activation function is especially noticeable for the case of a superposition of basic states. We have also shown how dissipation suppresses “quantum” oscillations on the activation function, just as damping suppresses plasma oscillations in classical Josephson systems. The obtained results pave the way for a classical perceptron and a control quantum co-processor (designed for the rapid search of the perceptron synaptic weights) to work in a single chip in a millikelvin cryogenic stage of a cryocooler. For the practical implementation of such neural networks, we need synapses, which are also based on adiabatic superconducting logic cells with magnetic representation of information [43,45,51]. Fortunately, there are already such elements based on an inductively shunted two-contact interferometer with the ability to adjust parameters. However, their behavior in the quantum mode requires an additional study.

## Funding

The *S*_Q_ neuron concept was developed with the support of the Russian Science Foundation (project no. 20-12-00130). The numerical simulations were supported by UNN within the framework of the strategic academic leadership program “Priority 2030” of the Ministry of Science and Higher Education of the Russian Federation. The work of AAG and AMS on the section ”Dynamics in a quantum neuron without dissipation” was carried out with the support of the RSF project no. 22-21-00586.

## Appendix 1

To solve Equation 6, we used the finite difference method [52], where a continuous wave function ψ(φ) is transferred to a discrete grid ϕ*_n_* = ϕ(φ*_n_*) with a step Δφ:


[22] $$-\left(\psi_{n+1}+\psi_{n-1}\right)+\left(2+\nu_{n}\right)\psi_{n}=\varepsilon_{n}\psi_{n}.$$

Here we introduce the following notations:


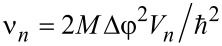


and


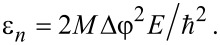


The boundaries ψ_0_ = ψ*_N_*_+1_ = 0 for Equation 22 are sufficiently removed from the region of actual motion of interest, and the wave functions of localized states are weakly affected by the introduced restrictions.

## Appendix 2

We have analyzed the evolution process on the basis of the Cayley algorithm [53]. The evolution operator of the system on a discrete time grid with a step Δ*t* is represented as:


[23]

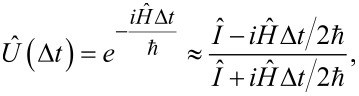



where 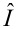 is the unit matrix corresponding to the dimensionality of the Hamiltonian of the system (Equation 5), 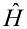, according to


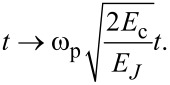


According to Equation 7, the Schrödinger time-dependent equation, and hence the dynamics of the system, can be found from the following equation:


[24] $$\psi_{n+1}^{j+1}=R_{n}^{j+1}\psi_{n}^{j+1}+S_{n}^{j+1},$$

where the auxiliary quantities are defined as


[25]

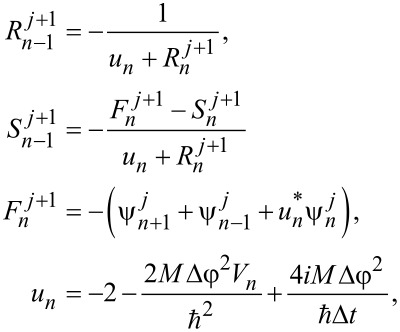



with boundary conditions


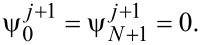


## Appendix 3

We will use only the Born–Markov approximation and neglect the Lamb shift. Hence, the generalized master equation [50] for the density matrix in terms of the instantaneous eigenbasis in the Schrödinger picture can be written as follows:


[26]

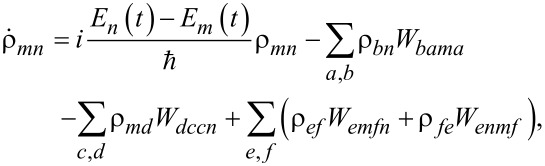



where the matrix elements *W_abcd_* are defined by


[27]

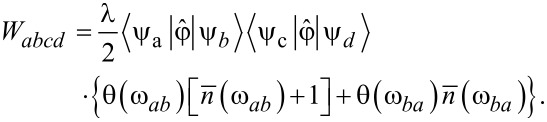



It should be noted that for Equation 26 it is necessary that the ground state and the first excited state are not nearly degenerate levels throughout the considered time of system evolution.

The generalized master equation with the secular approximation can be easily obtained from Equation 26 by multiplying the fourth term with the Kronecker delta symbol δ*_mn_* and by imposing additional conditions on the indices of summations, that is, *b* = *m*, *d* = *n* and *e* = *f*. Further, keeping only the diagonal terms of the density matrix, the Pauli master equation can be obtained. For all parameters, we have considered that the secular approximation has a negligibly small effect on the numerical solution of the generalized master equation.

**Figure 11:**
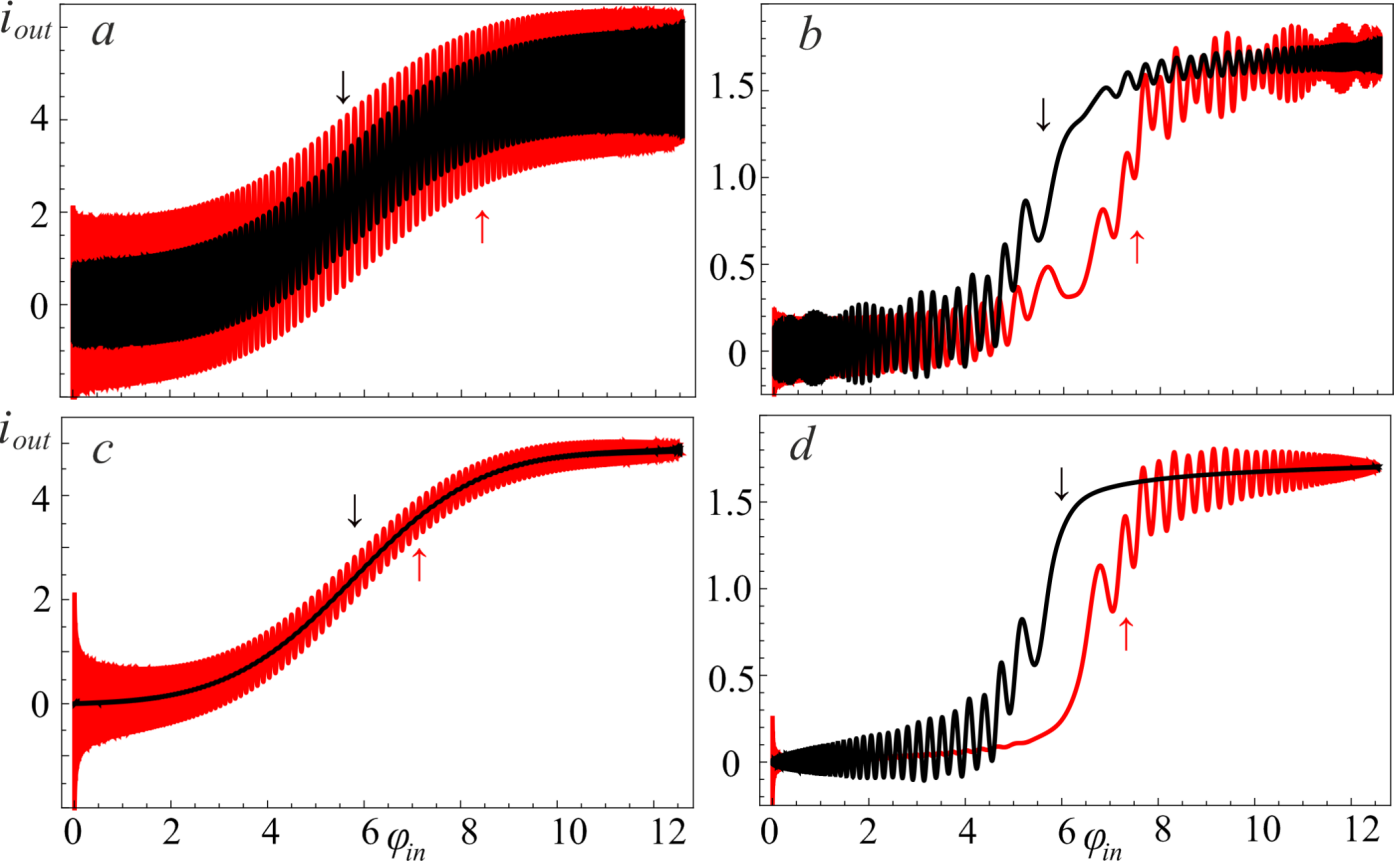
The neuron activation functions for *l* = 0.1 (a, c) and *l* = 2.5 (b, d) for different renormalized coupling constants: λ = 0.005 (a, b) and λ = 0.035 (c, d). The input flux parameters are as follows: *D* = 0.008, *A* = 4π, *t*_1_ = 500; the temperature of the bosonic thermostat is *T* = 50 mK; the cell is initialized in the superposition of the two basic states.

In Figure 11a–d, we present the activation functions obtained by solving the generalized master equation for different values of the inductance *l* and the renormalized coupling constant λ. Initial conditions are the superposition of states, that is, 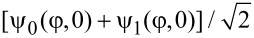. As expected, oscillations arise due to interference between levels, which decrease with increasing the coupling constant.

Note that for small l < l^*^, and for the neuron initialized either in the ground state or in the excited state, the solution of the generalized master equation is the same as the solution of the Pauli master equation.
